# The Therapeutic Value of the X-Ray in Lupus Vulgaris1Read at the thirty-fourth annual meeting of the Medical Association of Central New York, held at Buffalo, N. Y., August 27 and 28, 1901.

**Published:** 1901-10

**Authors:** Clarence A. Greenleaf

**Affiliations:** Rochester, N.Y.


### The Therapeutic Value of the X-ray in Lupus Vulgaris.[1]

By CLARENCE A. GREENLEAF, M. D., Rochester, N. Y.

DURING December of the year 1895, Prof. Röntgen announced the discovery of the *x*-ray. This year, 1901, Dr. Niels Finsen, of Copenhagen, was awarded one of the Noble prizes for his discovery of the light treatment for lupus, this being one of five prizes awarded annually to those who have done the most "for the benefit of humanity" during the year.

Tuberculosis, in its different manifestations, has always been a dreaded disease and generally considered incurable, and tuberculosis of the skin (lupus vulgaris) treated either medically or surgically, is one of the most difficult conditions we have to contend with.

During the past two years I have used the *x*-ray treatment for lupus vulgaris and report in detail the following four cases:

Case I.—Referred by Dr. Richard Moore, of Rochester, N. Y., July 1, 1900. L. M., female; aged 19. Born in Ireland. Came to the United States during June, 1899. Father, mother, three brothers and three sisters healthy. One cousin died of epithelioma of the lip. The patient states that when she was seven years of age she fell, injuring her upper lip and that the gum was sore for some time. About two years ago her nose began to bleed occasionally and was sore. Under the advice of a physician the part was poulticed with no beneficial result. She then consulted another physician, who cauterised the infected lip and nose, as she states, about two hundred times. The nose kept getting worse, bled more freely and the nostrils were finally occluded. Just previous to coming to America she entered a hospital in Dublin where the nose and upper lip were thoroughly curetted. After coming to this country she had no medical attendance until six months ago. During the interval the nose gradually became worse. During the past six months Dr. Moore has curetted five times with little improvement.

Examination, July 1, 1900, revealed an hypertrophy of the nose to about one-third more than its normal size, being of a dull reddish color. The skin was tense and doughy to the touch underneath, the entire tissue over the nasal bones being involved. Both nostrils were occluded, their mucous membranes discharging and hemorrhagic. The left ala of the nose was ulcerating for about one-half inch upon its outer surface. The mucous membranes and the ulcer bled at intervals and the patient suffered from headaches. The upper lip contained a few small nodules and cicatricial scars.

---

1. Read at the thirty-fourth annual meeting of the Medical Association of Central New York, held at Buffalo, N. Y., August 27 and 28, 1901.

A mask of sheet lead, one-eighth inch in thickness, was made and an opening cut large enough for the nose and upper lip to be exposed. A low vacuum $x$-ray tube was placed ten inches distant from the skin surface and an exposure of fifteen minutes given. From this date, with the exception of two weeks, those of September 5, 1900, and October 1, 1900, until the middle of November, three such exposures were made weekly. After the third treatment the hemorrhages and headaches stopped, and November 15th the right nostril was free. The left nostril while free from discharge was still occluded. The ulcer was healed and the hypertrophy had steadily diminished. From November 15, 1900, to January 1, 1901, two treatments were given, the condition remaining about the same. During January, 1901, the patient received two treatments weekly with little improvement, the parts remaining in about the same state with an occasional nose bleed. One small surface continued hard, but there was no ulceration or inconvenience. The patient was then given a rest for ten weeks. Examination after this period revealed no change. Two treatments a week were given until June, 1901, these producing a slight improvement in the appearance. The occlusion of the right nostril was due to hypertrophy of the turbinated bones and I was undecided as to the safety of removal, fearing a latent process might be stirred up. However I advised their removal and this was done June 13, 1901, by Dr. J. M. Ingersoll, at the Rochester City Hospital. There was considerable hemorrhage, but this soon stopped and the wound healed quickly. Since then four treatments have been given. Both nostrils are now free, but the mucous membrane is slightly inflamed and there is a small area of hardness in the right ala of the nose.

Case II. Came to the Out-patient Department of the Rochester City Hospital, October 18, 1900:

S. K., female, aged 32; family history negative; husband tubercular. Always healthy previous to one year ago, when present trouble began. This patient had received no treatment. One year ago a single hard nodule appeared upon the left cheek. This increased in size and resulted in an ulcer about the size of a lead pencil. During the year she has had four ulcers, varying in size, all upon the right cheek. At present the patient has cicatricial scars at the site of the first ulcers, also a large reddish patch, about the size of a half dollar, which she states was the last to heal upon the right side. Upon the left cheek are two open ulcers, about the size of ten and five-cent pieces respectively, the one upon the side of the nose, the other upon the cheek. These are nearly confluent and the surrounding zone of skin is red in color. Both ulcers are open with no crusting, being typical lupus ulcers.

The patient suffers no discomfort except a "drawing sensation." A lead mask was made conforming the openings to the size of the ulcers and the same methods employed as in Case I.

with the exception that in this case a higher vacuum tube was used. The first treatment was given October 12, 1900, and I did not see the patient again until November 5, 1900. The smaller patch on the left side was at this time crusted over. Upon the right side, just above one of the old scars a small nodule was present. This was hard and felt like a kernel of corn under the skin. This the patient stated, was the way all the ulcers started. An exposure was made at this time. The next exposure was made November 12, 1900. No change was apparent in the nodule, except that it was softer in feeling. November 14, 1900, the patient came to the office to be photographed and the nodule had entirely disappeared, leaving the skin in a normal condition. From this time until the middle of December, 1900, nine treatments were given. The patient was discharged December 15, 1900, the lupus patches entirely healed and the evidence of normal skin unquestionable. I saw this patient about four weeks later and the skin, with the exception of the irregular cicatrix was soft and normal.

Case III. Referred by Dr. William L. Conklin, of Rochester, N.Y., March 20, 1901:

Mrs. S., age 52; born in the United States; family history, tubercular. Mother died of consumption. One sister suffering from pulmonary tuberculosis at present. The patient states that in 1895 a hard lump appeared upon the inner side of the right thigh, resulting in an ulcer which remained open for four months. A few weeks later a similar condition occurred upon the outer side of the thigh. Carbolic solutions and iodine ointment were used, followed by milder ointments. From the outer side of the thigh the trouble passed down the limb to the outside of the knee. As soon as this closed it broke out over the kneecap. This occurred in the fall of 1897. This ulcer continued more or less active until last spring, when it closed for about five weeks and then reopened. The condition had spread down to the front part of the leg, about eight months before and this had continued open to the present time. The patient was treated at the Homeopathic Hospital for fourteen weeks, the ulcers being repeatedly cauterised with no improvement.

The limb shows cicatricial scars at the site of the two ulcers upon the outer and inner sides of the thigh. This same condition is present in the cicatrix outside of the knee. Directly over the anterior surface of the knee is a large ulcer three and one quarter by three and one half inches in diameter. Its edges suppurating and its center more or less hemorrhagic. Three and one quarter inches below this ulcer, upon the anterior surface of the leg, was another ulcer two by three and one-quarter inches in diameter. The center of this was deep, not only involving the skin, but the subcutaneous tissue.

I advised daily treatment for a time and the patient entered the Rochester City Hospital, March 23, 1901. The ulcers were exposed to the *x*-ray daily for two weeks. The patient then left the hospital and came to the office three times a week for treatment. This continued without intermission for six weeks. Since then she has had an occasional treatment. After the fifth treatment the knee ulcer crusted over and remained so, except when the removal of the dressing tore it open. Occasionally a slight discharge occurred from near the edge. The lower ulcer improved in appearance and contracted some, but was discharging still when the last treatment was given July 2, 1901.

I received no report from Mrs. B. during July and August, and called at her home August 24, 1901. She had been unable to leave her house since July 1, because at that time she had contracted a severe cold, which had affected her entire system. Severe pain in her leg had been relieved by poulticing, which softened the crusts, so that the ulcers were again open. Antiseptic washes had been used and the ulcers appeared more healthy. The upper ulcer had contracted slightly and the subcutaneous tissue of the lower ulcer had entirely filled in so that it was superficial.

Case IV.—Referred by Dr. E. Wood Ruggles, of Rochester, N.Y., August 6, 1901:

M. M., female; aged 24. Born in the United States of German parentage. No tubercular history can be obtained. About five years ago a small lump appeared upon the right arm which resulted in an ulcer. This healed with simple treatment. The cicatrix is about one by three inches in diameter. The patient states that about three years ago a small sore appeared upon the chin. This increased in size rapidly. About three weeks later a similar condition appeared upon the left cheek in front of the ear. The edges of this were cauterised three times and it healed promptly. The patch upon the chin received the same treatment and this has been continued for the past two years, but it has gradually spread until it now involves the entire chin and lower lip. It presents one large inflamed surface with several active nodules, the lip and its adjacent surface being ulcerating and painful. Treatments have been given three times a week. After the second treatment the exudate stopped in the upper part of the ulcer and crusting rapidly followed, the ulcer assuming a more healthy condition.

The benefit derived from the study of a series of cases determines the value of the treatment used. The importance of a prognosis in any case is manifest, and when a patient suffering from lupus desires to know what hope there is for ultimate recovery, it is our duty to inform this patient what we can do to relieve the condition. We have in this series, one case of direct tubercular infection, viz.: from the husband to the wife; another, a case of general systemic tubercular infection in which the lupus is prominent and two cases in which we are unable to trace the source. In three cases all known treatment, with the exception of complete excision, the light treatment and the $x$-ray treatment had been used. Excision seems out of the question when we consider the areas involved.

Dr. Finsen in his employment of the light treatment exposes the infected skin to a concentrated light derived from powerful arc lights or sunlight. He gives daily treatments of one hour duration. With the advantages he has in his large institute such methods are feasible. However, by the employment of the $x$-ray treatment, we have at our disposal an apparatus easily obtained. The patients are subjected to fifteen minutes treatments only, surely an advantage over hour sittings, and the results so far are as satisfactory as from the action of the $x$-ray.

Now in regard to the effect of the $x$-ray treatment upon these cases: The action of the light was the same in each case, viz., an immediate crusting over of the infected surface, a "drying up" so to speak. This was accompanied by the alleviation of other symptoms. The case which had received no previous treatment rapidly recovered, and in this patient the light had a remarkable effect upon a tubercular node, rendering it inert. The case which had received active treatment for so long a time improved rapidly, and while we cannot consider the patient completely recovered, however the symptoms are alleviated and the infected parts have assumed a normal appearance. I do not consider the third case indicative of failure in the treatment of these cases, because a more general element entered into the case, viz.: that of reinfection from the patient's systemic condition. The last case was included to show that so far in the treatment the same conditions of repair exist as occurred in the other cases.

From the observation of these cases, I wish to offer the following points: (1) That the most satisfactory treatment for lupus vulgaris is the $x$-ray treatment; (2) that repair begins almost immediately; (3) that the danger attending the treatment is minimum; (4) that the surroundings and general condition of the patient are an important element in the ultimate result; (5) that cases which have not been subjected to surgical treatment react rapidly and ultimately recover, whereas cases which have received surgical treatment react in a degree of slowness dependent upon the extent of previous surgical interference.

---

THE Philippine Commission has authorised the establishment of a government biological and chemical laboratory at Manila.

